# Interleukin-6 as a Milk Marker of Clinical and Subclinical Intramammary Infections (IMI) in Cows Caused by *Streptococcus* spp.

**DOI:** 10.3390/ani14071100

**Published:** 2024-04-04

**Authors:** Mariola Bochniarz, Monika Ziomek, Marek Szczubiał, Roman Dąbrowski, Marco Wochnik, Łukasz Kurek, Urszula Kosior-Korzecka, Aneta Nowakiewicz

**Affiliations:** 1 Sub-Department of Veterinary Microbiology, Faculty of Veterinary Medicine, University of Life Sciences in Lublin, Akademicka 12, 20-033 Lublin, Poland; aneta.nowakiewicz@up.lublin.pl; 2 Department of Food Hygiene of Animal Origin, University of Life Sciences in Lublin, Akademicka 12, 20-950 Lublin, Poland; monika.ziomek@up.lublin.pl; 3 Department and Clinic of Animal Reproduction, Faculty of Veterinary Medicine, University of Life Sciences, Gleboka 30, 20-612 Lublin, Poland; marek.szczubial@up.lublin.pl (M.S.); roman.dabrowski@up.lublin.pl (R.D.); marco.wochnik@up.lublin.pl (M.W.); 4 Department and Clinic of Animal Internal Diseases, Faculty of Veterinary Medicine, University of Life Sciences, Gleboka 30, 20-612 Lublin, Poland; lukasz.kurek@up.lublin.pl; 5 Department of Preclinical Veterinary Sciences, Faculty of Veterinary Medicine, University of Life Sciences in Lublin, Akademicka 12, 20-033 Lublin, Poland; urszula.korzecka@up.lublin.pl

**Keywords:** mastitis, *Streptococcus* spp., immune response, Interleukin-6

## Abstract

**Simple Summary:**

Mastitis is an inflammatory reaction of the udder tissue in the mammary gland, caused by microbial infection. It is considered the most common disease in a herd of dairy cows. Etiological agents include a variety of Gram-positive and Gram-negative bacteria, fungi, even algae. Data from the literature indicate that among the pathogens of mastitis, *Streptococcus* species, currently predominate which was also confirmed in our study. *Streptococcus* spp. can cause both a subclinical form of mastitis and one that develops with strongly expressed clinical symptoms. The body’s response to infection is an inflammatory response and rapid production of acute-phase proteins. Our earlier studies showed a significant increase in the concentration of amyloid A in the milk of cows suffering from streptococci-induced mastitis. The main inducer of acute-phase proteins is interleukin-6. This cytokine is secreted by various cells, including lymphocytes, monocytes, macrophages and endothelial cells. IL-6 activates innate and adaptive immune responses, recruits immune cells and triggers B- and T-cell responses. Monitoring of IL-6 levels in milk can allow early detection of mastitis, which is especially important in cases of subclinical inflammation.

**Abstract:**

The aim of the study was to evaluate the concentrations of Interleukin-6 (IL-6) in milk and serum of healthy cows (HE) and cows with mastitis caused by *Streptococcus* spp. The blood and milk samples were obtained from Holstein-Friesian cows (Lublin region, Poland). A total of 43 milk and serum samples from 28 cows with mastitis and 15 healthy cows were selected for study. IL-6 levels in milk from HE cows ranged from 6.09–80.24 pg/mL (median 26.6 pg/mL) and were significantly lower than in milk from both cows with clinical and subclinical mastitis (487.09 pg/mL vs. 26.6 pg/mL in CM, *p* < 0.001; and 165.31 pg/mL vs. 26.6 pg/mL in SCM, *p* < 0.001). The IL-6 concentration in the serum of HE was not significantly different from the serum IL-6 of the entire group of mastitis cows, regardless of whether the inflammation proceeded in a clinical or subclinical form (44.37 pg/mL vs. 78.09 pg/mL; 128.29 pg/mL vs. 78.09 pg/mL, respectively). The present study indicates that cows with mastitis caused by *Streptococcus* spp. develop a local immune response in the mammary gland in response to the pathogen. Monitoring of IL-6 levels in milk can allow early detection of mastitis, which is especially important in cases of subclinical inflammation.

## 1. Introduction

Mastitis (intramammary infection) describes inflammation of the udder tissues due to microbial infection. It is considered the most common disease in a herd of dairy cows, which is associated with high costs due to a decrease in milk production and the need to exclude treated cows from milk production [1,2,3,4]. The reduction in milk production due to tissue damage in the udder accounts for 70% of total losses [5]. The owner also must cover the costs associated with mastitis diagnosis and treatment. In case of therapeutic failure, the cow should be removed from the herd. Improving milk-production hygiene, such as more-frequent milking practices, implementing teat disinfection, and maintenance of milking equipment are general steps to prevent new cases of mastitis, particularly those caused by infectious pathogens [6,7].

Mastitis can be caused by various pathogens (Gram-positive and Gram-negative bacteria, fungi, and even algae). Data from the literature indicate that among the pathogens of mastitis, *Streptococcus* species currently predominate [2,8,9,10].

Bovine mastitis caused by *Streptococcus* spp. can occur in clinical (CM) and subclinical (SCM) forms, and with a longer process, sometimes progresses to a chronic form [2,11,12]. In the course of clinical mastitis, the cow has visible, easily detectable symptoms such as redness, swelling, and soreness of the udder and in some cases also fever and lack of appetite. Milk from the diseased quarter of the udder has a watery consistency with the presence of flakes and clots [13,14]. Typically, the disease develops very quickly, and symptoms appear suddenly. Clinical mastitis, depending on the severity of symptoms and the course of the process, can be divided into peracute, acute, and subacute [5,11,14]. In subclinical mastitis, there are no visible symptoms in the udder or milk with a simultaneous increase in the somatic cell count (SCC) above 200,000/mL and a decrease in milk production [5,7,13,15]. Losses due to subclinical mastitis are definitely higher than in the clinical form of mastitis although very difficult to estimate [16,17]. According to Shearer and Harris [18], subclinical mastitis appears far more often than clinical forms (15 to 40 times), and moreover, the process is dangerous because it can progress to clinical forms. The occurrence of subclinical inflammations is very dangerous for the herd because they are difficult to detect, last a long time, and adversely affect milk quality and production. On the other hand, chronic mastitis lasts for up to several months, with the possibility of clinical acute symptoms at irregular intervals. In a large number of cases, infection of the udder by *Streptococcus* spp. causes irreversible changes including hypertrophy of the walls of the sinuses and milk ducts and atrophy of the secretory tissue resulting in permanent loss of milk production capacity [2,6].

The response of the cow’s body to infection is the rapid production of so-called acute-phase proteins (APPs), among which the most important are serum amyloid A (SAA), haptoglobin, and complement components [19,20,21]. They are involved in killing microorganisms and removing cells, while protease inhibitors inactivate hydrolases released from immune cells, which protects tissues from damage [19,20,21,22]. IL-6 is one of the most important factors inducing the production of APPs by hepatocytes in the liver [23,24,25]. The cytokine gene expression of bovine IL-6 has been demonstrated in normal mammary glands and in mastitis. As a pro-inflammatory cytokine, it activates innate and adaptative immune responses, recruits immune cells, and triggers B and T cell responses [23,26,27]. IL-6 is one of the pro-inflammatory cytokines responsible for the symptoms of acute septic shock in mastitis caused by coliform bacteria [28,29]. During mastitis in cattle, CD4+ T lymphocytes, activated upon antigen recognition, predominate. IL-6 is produced by Th2 cells [27].

The aim of the study was to evaluate the concentrations of IL-6 in serum and milk from cows in the course of clinical and subclinical mastitis caused by *Streptococcus* spp.

## 2. Materials and Method

Blood and milk samples were taken from Holstein-Friesian cows from a single herd in Lublin Province, Poland. The selected herd was one in which documentation and medical history confirmed a problem with udder infections in cows by *Streptococcus* spp. running in both clinical and subclinical forms. The herd consists of 126 lactating cows and 22 dry cows. The cows are housed in a tie stall with no possibility of going out to pasture. They are fed on a total-mixed-ration system (full-feed, year-round TMR). The mean milk productivity was 7336 kg per cow per year. Milking was carried out twice a day.

Quarter milk samples (QMS) were routinely collected before morning milking from all lactating cows that were not receiving any drugs at the time, for the California mastitis test (CMT). Prior to milk collection, each cow was clinically examined. The main attention was focused on the exploration of the udder. A thorough palpation examination was performed to determine inflammatory swelling, fibrosis, induration, and other clinical signs. Milk samples were also evaluated macroscopically (visual examination for changes in color and consistency and the presence of admixtures such as blood). This study was performed in the Schalm disc test with four wells, just preceding the performance of CMT (Mastirapid, Vetoquinol Biowet, Poland). The results of the CMT were estimated as negative (0), trace (T), weakly positive (1+), distinctive positive (2+), and strongly positive (3+), as indicated by Quinn et al. [30]. Milk was aseptically collected from each udder quarter with a positive CMT result. First, the udder was washed and dried and the teat apex was disinfected with 70% alcohol. Next, the first three streams of milk were discarded, and the subsequent flows of milk were collected into three sterile containers (two containers—40 mL and one tube—10 mL). In addition, blood was collected from each CMT-positive cow in at least one quarter of the udder. Samples were taken from the external jugular vein into a tube containing an anticoagulant (ethylenediaminetetraacetic acid (EDTA)). A second blood sample was collected into tubes containing a serum clotting activator (Medlab, Raszyn, Poland).

The material for laboratory testing (milk and blood samples) was delivered at under 4 °C in less than 2 h. One sample of fresh milk (approximately 40 mL) was used for somatic cell count (SCC) testing using a SomaCount FC Automatic (Bentley Instruments Inc., Chaska, MN, USA). A second sample of fresh milk was used for bacteriological testing. In turn, the third milk sample was intended to measure IL-6 levels. Both milk and blood samples were then centrifuged at 1000× *g* for 20 min. The obtained blood serum and milk whey were transferred into Eppendorf tubes and frozen until testing. From the blood sample collected into the tube containing EDTA, the levels of red and white blood cells (RBC and WBC), hemoglobin (HGB), and hematocrit (HCT) were tested using the Scil ABC+ Vet Animal Hematology Analyzer (Horiba, Kyoto, Japan). The results of this study are shown in Table 1.

### 2.1. Bacteriological Examination

Columbia agar (Oxoid, Basingstoke, UK) and Edwards medium (Oxoid, Basingstoke, UK), both supplemented with 5% dehydrated sheep blood, were used for bacteriological testing. Milk was inoculated using a 0.01 mL loopful. Plates were incubated under aerobic conditions for 24 h at 37 °C. Gram-stained microscope slides were made of the cultured bacterial colonies. Streptococci are Gram-positive bacteria that are spherical in shape (0.5–2 µm) and usually form pairs or chains. *S. agalactiae* and *S. dysgalactiae* grew as blue, gray, or colorless, transparent colonies. Induction of β-hemolysis was demonstrated using the CAMP assay (Christie, Atkins, Munch-Petersen). *S. uberis* and enterococci hydrolyzed esculin to glucose and esculetin and grew dark brown colonies on Edwards medium. Cultures with the ability to degrade esculin were inoculated on Kanamycin Esculin Azide Agar (Oxoid, Basingstoke, UK), to distinguish streptococci from enterococci. In addition, the biochemical characteristics of the bacteria, such as the production of catalase, were analyzed. Only Gram-positive, catalase-negative granules whose colony appearance and biochemical characteristics were indicative of *Streptococcus* spp. were included for further study. Final identification was carried out using a matrix-assisted laser desorption/ionization—time of flight mass (MALDI TOF) system (Bruker Daltonics, Bremen, Germany).

### 2.2. Study Groups

Seventy-six quarter milk samples of the 504 tested were positive for CMT. Cows showing signs of other associated diseases, including metabolic diseases, were not included in the study. The remaining cows were divided into 2 groups: CM (clinical mastitis) and SCM (subclinical mastitis), according to the type of mastitis. The defining feature of SCM cows was the absence of visible signs of udder infection but the presence of microorganisms in the milk was confirmed at SCC > 200,000/mL of milk. Any case in which various milk and udder abnormalities were visible (clots and flakes in the milk, swelling, redness, or pain in the udder) along with elevated SCC > 200,000/mL of milk was included in the CM group. The next step in including cows in the study was the result of a bacteriological test. The decisive criterion was the isolation of *Streptococcus* spp. from the milk of sick cows.

Twenty-eight cows with streptococcal mastitis (16 with SCM and 12 with CM) were included in the study. Milk and blood samples were also taken from 15 healthy cows with no apparent signs of the disease, which were negative on the milk CMT test. These cows were included in the control group. The health status of all cows was confirmed by hematological examination (Table 1). All procedures for collecting material for animal testing carried out within the project were recognized by the Local Ethical Committee for Animal Experiments in Lublin as routine veterinary services for dairy cows. Therefore, the study was conducted in accordance with Polish law (Act of 17 November 2021 amending the Act on the protection of animals used for scientific or educational purposes (Dz.U. 2021, poz. 2338), as well as with European Union regulations contained in the Directive 2010/63/EU on the protection of animals used for scientific purposes.

### 2.3. Measurements of IL-6 in Serum and Quarter Milk Samples

The concentrations of cytokines in blood serum and quarter-milk samples were determined by the ELISA method using kits for IL-6 from USCN Life Science Inc., Houston, Texas, USA. All procedures were performed according to the guidelines provided by the manufacturers. Absorbance readings were performed on an automatic microtiter plate reader (ELx800, Biotek Instruments, Winooski, VT, USA) at 450 nm using 630 nm as a reference. The detection range of IL-6 for cattle was 1.56–100 pg/mL. The inter- and intra-assay coefficients of variation (CV) for all examined cytokines were <12% and <10%, respectively.

### 2.4. Statistical Analysis

The present study compared the levels of IL-6 in the serum and milk of cows with clinical and subclinical mastitis caused by *Streptococcus* spp. with levels in healthy cows. First, the Shapiro–Wilk test was applied to determine/exclude the normality of the distribution of trait values in the study groups. Then, the Mann–Whitney test was performed for two independent samples. *p* < 0.05 was considered significant. Statistica 12.0 statistical package (StatSoft. Inc., Tulsa, OK, USA) was used to perform the calculations.

## 3. Results

The study was conducted on 28 samples of milk and 28 samples of serum from cows with subclinical and clinical mastitis caused by *Streptococcus* spp. and 15 samples of milk and 15 samples of serum from healthy cows. *Streptococcus* species found in the cows were: *Streptococcus dysgalactiae* (10 isolates), *Streptococcus uberis* (12), *Streptococcus agalactiae* (4 isolates), and *Streptococcus canis* (2 isolates). The SCC in the milk of these cows ranged from 324,000–2,680,000 cells/mL. One milk sample and one serum sample from each cow were qualified for evaluation of IL-6. The results of the study are provided in Table 2 and Table 3.

IL-6 levels in milk from healthy cows ranged from 6.09–80.2 pg/mL (median 26.6 pg/mL) and were significantly lower than in milk from all cows suffering from mastitis caused by *Streptococcus* spp. (82.66–695.61 pg/mL with median 344.21 pg/mL, *p* < 0.001). It should be noted that a significant difference in concentration of IL-6 in the milk compared to the control group was recorded for both cows with clinical and subclinical mastitis (487.09 pg/mL vs. 26.6 pg/mL in CM, *p* < 0.001; and 165.31 pg/mL vs. 26.6 pg/mL, *p* < 0.001). However, there was no statistically significant difference in IL-6 milk concentration between the CM and SCM groups (487.09 pg/mL vs. 165.31 pg/mL) (Table 2 and Table 3).

The IL-6 concentration in the serum of healthy cows ranged from 74.1–199.62 pg/mL and was not significantly different from the serum IL-6 of the entire group of mastitis cows (106.3 pg/mL vs. 78.09 pg/mL), regardless of whether the inflammation proceeded in a clinical or subclinical form (44.37 pg/mL vs. 78.09 pg/mL; 128.29 pg/mL vs. 78.09 pg/mL, respectively). There was also no statistically significant difference in serum IL-6 levels between the CM and SCM groups (44.37 pg/mL vs. 128.29 pg/mL) (Table 2 and Table 3).

The results of the study indicate that the content of IL-6 in the milk of mastitis cows was significantly lower than that in the serum of healthy cows (26.6 pg/mL vs. 106.3 pg/mL, *p* < 0.001). Moreover, the content of IL-6 in the milk of cows with CM was significantly higher than that in the serum of cows with CM (487.09 pg/mL vs. 44.37 pg/mL, *p* = 0.003, respectively). Only in the group of cows with the subclinical form of mastitis, were the IL-6 levels in serum and milk at similar values (128.29 pg/mL vs. 165.31 pg/mL) (Table 3).

## 4. Discussion

The goal of the study was to investigate changes in the level of IL-6 in milk and serum of cows with clinical and subclinical streptococci-induced mastitis compared to healthy cows.

Due to its many important functions during inflammation, such as host defense through the immune response, hematopoiesis, and regulation of inflammation, Il-6 is one of the most important pro-inflammatory cytokines [26,27]. It is the main inducer of hepatic induction of acute-phase proteins during infection [1,23,24,25,27]. Pathogens penetrating the udder stimulate various cells, including lymphocytes, monocytes, macrophages, endothelial cells, epithelial cells, and fibroblasts, to secrete this cytokine.

IL-6 has been recognized as an early but non-specific indicator of mastitis [24,27,31]. Increased concentrations of IL-6 in milk and blood were detected by the authors in cows and in ewes with natural infection of the udder but also in the case of experimentally induced mastitis and even after administration of bacterial lipopolysaccharides (LPS) [24,26,28,32]. Previous studies indicate that the level of IL-6 depends on a number of factors, including the form of mastitis course, the phase of infection, and the type of microorganisms causing mastitis [26,27,31]. Hagiwara et al. [26] showed a significant increase in IL-6 levels during the initial phase of infection of the udder caused by *E. coli*, *K. pneumoniae*, *S. aureus*, and *Streptococcus* spp., but significantly lower in the case of coagulase-negative staphylococci.

The present study also showed significantly higher concentrations of IL-6 in the milk of cows with streptococci-induced mastitis compared to the milk of healthy cows, with markedly elevated levels of this cytokine recorded in the milk of cows with mastitis occurring in clinical form compared to those with the subclinical form (18 and six times higher than in HE, respectively). In contrast, our earlier study on mastitis in cows with subclinical coagulase-negative staphylococcal mastitis was not consistent with the results of Hagiwara et al. [26] study because it showed significantly higher concentrations of IL-6 both in milk (20 times) and in serum (2.5 times) compared to healthy cows [31]. Considerably increased milk levels of IL-6 in cows with staphylococci-induced mastitis were also recorded by Osman et al. [23] with the noteworthy fact that these values were higher in the subclinical form than in the clinical form of mastitis. On the other hand, Shaheen et al. [12] showed a significant increase in IL-6 levels in cows with clinical mastitis while, in cows with SCM, the increase in this cytokine was statistically insignificant compared to HE. Also, studies conducted on bovine mammary epithelial cells (MEC) indicate a rapid response to infection of the udder by pathogens expressed by IL-6 secretion. Secretion of pro-inflammatory cytokines and antimicrobial molecules (cathelicidin, defensins) begins after recognition of microbial components by epithelial cells in the udder [33,34]. IL-6 levels in bovine MEC increased at 6 and 24 h after experimental infection with *S. aureus* [13]. Moreover, Günther et al. [35] found that IL-6 is the only cytokine showing increased mRNA expression in MEC after *S. aureus* infection. Increased levels of pro-inflammatory cytokines are an extremely important response of the body to infection, as they initiate the mobilization of neutrophils to the site of infection in the udder [36]. These pro-inflammatory cytokines play a key role in fighting the primary infection [24,28]. Studies by a number of authors support the thesis that IL-6 can be considered a potential marker of mastitis, which is especially important when the subclinical form is difficult to notice. Detection of high concentrations of IL-6 in milk samples from cows affected by subclinical mastitis allows the detection of inflammation earlier than SCC [24].

Our study confirmed an elevated concentration of IL-6 in the milk of unhealthy cows. However, there was no notable change in IL-6 content in the serum of diseased cows in comparison with the control group, which may mean that the process was local in the mammary gland. It should also be noted that in the group of cows suffering from CM, the level of IL-6 in serum did not differ in value from the serum of healthy cows, which means that the increased inflammatory process in the mammary gland did not affect the systemic immune response. However, it is important to note the varying levels of IL-6 in the milk and serum of both healthy cows and cows with CM. In the milk of healthy cows, the level of IL-6 was four times lower in milk than in serum, while in the group of cows with CM, it was 3.7 times higher in milk than in serum. The results of this study clearly show that the mammary gland is the main point of defense against pathogens. Moreover, in addition to the local response to infection, there is an increased flow of defense cells from the peripheral blood to the diseased quarter of the udder and, due to this, an elevated level of pro-inflammatory cytokines is activated.

Song et al. [37] concluded that an incredibly valuable advantage of IL-6 is also its ability to stay in the blood circulation for longer times than other proinflammatory cytokines. A study by Vitenberg et al. [27] showed an elevation in the average number of cells immunoreactive for IL-6 in the milk of cows with CM from 9.6 cells on the fourth day, to 16.2 cells on the fifth day, to 17.2 cells on the sixth day of the experiment. As the inflammatory process continues, IL-6 levels decrease. Kawecka-Grochocka et al. [38] found lower IL-6 levels in cows suffering from chronic mastitis caused by coagulase-positive staphylococci compared to healthy cows while finding no difference in IL-6 gene mRNA levels. This can suggest that during chronic mastitis, the host stops producing proinflammatory cytokines, most probably to protect host tissues from being damaged during long-term infection [38]. It should, therefore, be emphasized that the impact of infection is dependent on the host reaction in the early phases of the disease, including the activity of released cytokines. The role of cytokines is to induce or inhibit numerous processes. Mastitis, whether occurring naturally or induced experimentally, causes an increase in the number of SCC and the level of cytokines produced in milk [27].

In conclusion, our results imply that cows suffering from mastitis induced by Streptococcus spp. develop a local immune reaction in the udder in response to the pathogen. We have noted an elevated concentration of proinflammatory cytokine—IL-6 in milk but not in serum, in both cows with clinical and subclinical mastitis. The monitoring of this protein in the milk of dairy cows could detect the subclinical inflammatory conditions of the mammary gland and, in this way, limit the occurrence of persistent mastitis and the spread of microorganisms in the environment.

## Figures and Tables

**Table 1:** Blood cell counts in cows with clinical (CM) and subclinical (SCM) mastitis caused by *Streptococcus* spp. (STR) and in healthy cows (HE).

	Clinical Mastitis	Subclinical Mastitis	HE
Blood WBC (×10^3^/mm^3^) *	8.24	7.80	8.02
lymphocytes (×10^3^/mm^3^) *	3.27	3.34	5.94
neutrophils (×10^3^/mm^3^) *	5.38	4.20	3.80
eosinophils (×10^3^/mm^3^) *	0.56	0.38	0.27
Blood RBC (×10^6^/mm^3^) *	6.43	6.84	7.80
HGB (g/dL) *	11.18	12.22	12.10
HCT (%) *	32	34	38

Legend: * average counts for all animals from each group. WBC—white blood cells, RBC—red blood cells, HGB—hemoglobin, HCT—hematocrit.

**Table 2:** The concentration of Interleukin-6 (IL-6) in milk and serum from healthy cows (HE) and cows with mastitis caused by *Streptococcus* spp. (STR).

Symbol	Sample	N		IL-6 pg/mL	
			Median	Min	Max
A	MILK HE	15	26.6 ^B,C^	6.09	80.24
B	MILK STR	28	344.21 ^A,D^	82.66	695.61
C	SERUM HE	15	106.3 ^A^	74.1	199.62
D	SERUM STR	28	78.09 ^B^	15.77	345.79

Legends: Data are presented as median, minimum, and maximum values. N—number of samples. Statistical analysis was performed using Mann–Whitney test (*p* < 0.05) vs. respective group indicated by symbol (^A–D^).

**Table 3:** The concentration of Interleukin-6 (IL-6) in milk and serum from healthy cows (HE) and cows with subclinical (SCM) and clinical (CM) mastitis caused by *Streptococcus* spp. (STR).

Symbol	Sample	N		IL-6 pg/mL	
			Median	Min	Max
A	MILK HE	15	26.6 ^B,C,D^	6.09	80.24
B	MILK SCM	16	165.31 ^A^	82.66	628.9
C	MILK CM	12	487.09 ^A,F^	133.91	695.61
D	SERUM HE	15	106.3 ^A^	74.1	199.62
E	SERUM SCM	16	128.29	16.17	276.42
F	SERUM CM	12	44.37 ^C^	15.77	345.79

Legends: Data are presented as median, minimum, and maximum values. N—number of samples. Statistical analysis was performed using Mann–Whitney test (*p* < 0.05) vs. respective group indicated by symbol (^A–D, F^).

## Data Availability

All compulsory data are presented in the manuscript, and we do not have any additional data.

## References

[1] Alluwaimi A.M. (2004). The Cytokines of Bovine Mammary Gland: Prospects for Diagnosis and Therapy. Res. Vet. Sci..

[2] Krömker V., Reinecke F., Paduch J.H., Grabowski N. (2014). Bovine *Streptococcus uberis* intramammary infections and mastitis. Clin. Microbiol..

[3] Bochniarz M., Błaszczyk P., Szczubiał M., Vasiu I., Adaszek Ł., Michalak K., Pietras-Ożga D., Wochnik M., Dąbrowski R. (2023). Comparative analysis of total protein, casein, lactose, and fat content in milk of cows suffering from subclinical and clinical mastitis caused by *Streptococcus* spp.. J. Vet. Res..

[4] Seegers H., Fourichon C., Beaudeau F. (2003). Review article: Production effects related to mastitis and mastitis economics in dairy cattle herds. Vet. Res..

[5] Cheng W.N., Han S.G. (2020). Bovine mastitis: Risk factors, therapeutic strategies, and alternative treatments—A review. Asian-Australas. J. Anim. Sci..

[6] Keefe G.P. (2012). Update on control of *Staphylococcus aureus* and *Streptococcus* agalactiae for management of mastitis. Vet. Clin. North Am. Food. Anim..

[7] Ruegg P. (2017). Mastitis detection, management, and prevention. J. Dairy Sci..

[8] Kabelitz T., Aubry E., van Vorst K., Amon T., Fulde M. (2021). The Role of *Streptococcus* spp. in Bovine Mastitis. Microorganisms.

[9] Bochniarz M., Szczubiał M., Brodzki P., Krakowski L., Dąbrowski R. (2020). Serum amyloid A as an marker of cow’s mastitis caused by *Streptococcus* sp.. Comp. Immunol. Microbiol. Infect. Dis..

[10] Taponen S., Liski E., Heikkilä A.M., Pyörälä S. (2017). Factors associated with intramammary infection in dairy cows caused by coagulase-negative staphylococci, *Staphylococcus aureus*, *Streptococcus uberis*, *Streptococcus dysgalactiae*, *Corynebacterium bovis*, or *Escherichia coli*. J. Dairy Sci..

[11] Cobirka M., Tancin V., Slama P. (2020). Epidemiology and Classification of Mastitis. Animals.

[12] Shaheen T., Bilal Ahmad S., Rehman M.U., Muzamil S., Razak Bhat R., Hussain I., Bashir N., Mir M.U.R., Paray B.A., Dawood M.A.O. (2020). Investigations on Cytokines and Proteins in Lactating Cows with and without Naturally Occurring Mastitis. J. King Saud. Univ. Sci..

[13] Khan M.Z., Khan A. (2006). Basic facts of mastitis in dairy animals: Review. Pak. Vet. J..

[14] Gruet P., Maincent P., Berhelot X., Kaltsatos V. (2001). Bovine mastitis and intramammary drug delivery: Review and perspectives. Adv. Drug Deliver. Rev..

[15] Abebe R., Hatiya H., Abera M., Megersa B., Asmare K. (2016). Bovine mastitis: Prevalence, risk factors and isolation of *Staphylococcus aureus* in dairy herds at Hawassa milk shed, South Ethiopia. BMC Vet Res..

[16] Romero J., Benavides E., Meza C. (2018). Assessing financial impacts of subclinical mastitis on colombian dairy farms. Front. Vet. Sci..

[17] Zhao X., Lacasse P. (2008). Mammary tissue damage during bovine mastitis: Causes and control. J. Anim. Sci..

[18] Shearer J.K., Harris B. (2003). Mastitis in Dairy Goats.

[19] Alnakip M.E., Quintela-Baluja M., Böhme K., Fernandez-No I., Caamaño-Antelo S., Calo-Mata P., Barros-Velázquez J. (2014). The immunology of mammary gland of dairy ruminants between healthy and inflammatory conditions. J. Vet. Med..

[20] Eckersall P.D., Young F.J., McComb C., Hogarth C.J., Safi S., Weber A., McDonald T., Nolan A.M., Fitzpatrick J.L. (2001). Acute phase proteins in serum and milk from dairy cows with clinical mastitis. Vet. Rec..

[21] Grönlund U., Hulten C., Eckersall P.D., Hogarth C., Persson K.W. (2003). Haptoglobin and serum amyloid A in milk and serum during acute and chronic experimentally induced *Staphylococcus aureus* mastitis. J. Dairy Sci..

[22] Kováč G., Tóthová C., Nagy O., Seidel H. (2011). Milk amyloid A and selected serum proteins in cows suffering from mastitis. Acta Vet. Brno.

[23] Osman K.M., Hassan H.M., Ibrahim I.M., Mikhail M.M. (2010). The impact of staphylococcal mastitis on the level of milk IL-6, lysozyme and nitric oxide. Comp. Immunol. Microb..

[24] Sakemi Y., Tamura Y., Hagiwara K. (2011). Interleukin-6 in quarter milk as a further prediction marker for bovine subclinical mastitis. J. Dairy Res..

[25] Scheller J., Chalaris A., Schmidt-Arras D., Rose-John S. (2011). The pro- and anti-inflammatory properties of the cytokine interleukin-6. Biochim. Et Biophys. Acta.

[26] Hagiwara K., Yamanaka H., Hisaeda K., Taharaguchi S., Kirisawa R., Iwai H. (2001). Concentration of IL-6 in serum and whey from healthy and mastitic cows. Vet. Res. Commun..

[27] Vitenberga-Verza Z., Pilmane M., Šerstņova K., Melderis I., Gontar Ł., Kochański M., Drutowska A., Maróti G., Prieto-Simón B. (2022). Identification of Inflammatory and Regulatory Cytokines IL-1α-, IL-4-, IL-6-, IL-12-, IL-13-, IL-17A-, TNF-α-, and IFN-γ-Producing Cells in the Milk of Dairy Cows with Subclinical and Clinical Mastitis. Pathogens.

[28] Shuster D.E., Lee E.K., Stevens M.G. (1993). Cytokine production during endotoxin-induced mastitis in lactating dairy cows. Am. J. Vet. Res..

[29] Riollet C., Rainard P., Poutrel B. Cells and Cytokines in Inflammatory Secretions of Bovine Mammary Gland. In Biology of the Mammary Gland; Mol, J.A., Clegg, R.A., Eds.; Advances in Experimental Medicine and Biology; Springer; Boston, MA, USA, 2002; pp. 247–258.

[30] Quinn P.J., Carter M.E., Markey B.K., Carter G.R. (2002). Bacterial causes of bovine mastitis. Veterinary Microbiology and Microbiol Disease.

[31] Bochniarz M., Zdzisińska B., Wawron W., Szczubiał M., Dąbrowski R. (2017). Milk and serum IL-4, IL-6, IL-10 and amyloid A (MAA, SAA) concentrations in cows with subclinical mastitis caused by coagulase-negative staphylococci (CNS). J. Dairy Sci..

[32] Pelayo R., Marina H., Suarez-Vega A., Gonzalo Hervas G., Esteban-Blanco C., Gausseres B., Foucras G., Arranz J., Gutierrez-Gil B. (2023). Influence of a temporary restriction of dietary protein in prepubertal ewe lambs on first lactation milk traits and response to a mammary gland inflammatory challenge. Res. Vet. Res..

[33] Boudjellab N., Chan-Tang H.S., Zhao X. (2000). Bovine Interleukin-1 Expression by Cultured Mammary Epithelial Cells (MAC-T) and Its Involvement in the Release of MAC-T Derived Interleukin-8. Comp. Biochem. Physiol. A Mol. Integr. Physiol..

[34] Bronzo V., Lopreiato V., Riva F., Amadori M., Curone G., Addis M.F., Cremonesi P., Moroni P., Trevisi E., Castiglioni B. (2020). The Role of Innate Immune Response and Microbiome in Resilience of Dairy Cattle to Disease: The Mastitis Model. Animals.

[35] Günther J., Esch K., Poschadel N., Petzl W., Zerbe H., Mitterhuemer S., Blum H., Seyfert H.M. (2011). Comparative kinetics of *Escherichia coli*- and *Staphylococcus aureus*-specific activation of key immune pathways in mammary epithelial cells demonstrates that *S. aureus* elicits a delayed response dominated by interleukin-6 (IL-6) but not by IL-1A or tumor necrosis factor alpha. Infect. Immun..

[36] Sordillo L.M., Shafer-Weaver K., DeRosa D. (1997). Immunobiology of the Mammary Gland. J. Dairy Sci..

[37] Song X., Wang T., Zhang Z., Jiang H., Wang W., Cao Y., Zhang N. (2015). Leonurine exerts anti-inflammatory effect by regulating inflammatory signaling pathways and cytokines in LPS-induced mouse mastitis. Inflammation.

[38] Kawecka-Grochocka E., Zalewska M., Rzewuska M., Kościuczuk E., Ząbek T., Sakowski T., Marczak S., Bagnicka E. (2021). Expression of cytokines in dairy cattle mammary gland parenchyma during chronic staphylococcal infection. Vet. Res..

